# Effects of different exercise types on body image in females: a systematic review and network meta-analysis

**DOI:** 10.3389/fpsyg.2026.1942447

**Published:** 2026-09-11

**Authors:** Jianglin Luo, Chen Lyu, Jiayu Niu, Zhaocheng Lin, Yanhua Shi, Zhili Ma

**Affiliations:** 1 College of Physical Education and Health, Guangxi Normal University, Guilin, China; 2 Faculty of Education, Guangxi Normal University, Guilin, China; 3 School of Physical Education and Health, Chengdu University of Traditional Chinese Medicine, Chengdu, China

**Keywords:** body image, exercise, females, network meta-analysis, systematic review

## Abstract

**Background:**

Negative body image is prevalent among women and is linked to emotional disorders, low self-esteem, and reduced quality of life. Exercise is an accessible non-pharmacological strategy that may improve body image, but the comparative effectiveness of different modalities remains unclear.

**Methods:**

PubMed, Embase, Web of Science, and the Cochrane Library were searched from inception to May 2026. Randomized controlled trials (RCTs) of exercise interventions lasting at least 4 weeks were included. Two reviewers independently conducted study selection, data extraction, and risk-of-bias assessment using the Cochrane Risk of Bias 2 (RoB 2) tool. A frequentist random-effects network meta-analysis was performed, with effects expressed as standardized mean differences (SMDs) and interventions ranked using P-scores.

**Results:**

Twenty-three trials involving 1,914 female participants and seven network nodes were included. Aerobic exercise, dance, and multimodal exercise significantly improved body image relative to usual care. Resistance training showed a favorable but imprecise effect, whereas mind–body exercise and behavioral intervention did not differ significantly from usual care. Aerobic exercise had the highest P-score, but confidence intervals for the leading interventions overlapped substantially and most active-treatment comparisons were not statistically significant. Network heterogeneity was moderate (I^2^ = 59%). Publication-bias assessments were discordant, and publication bias could not be excluded.

**Conclusion:**

Exercise appears to be a useful strategy for improving body image in women. Aerobic exercise and dance showed the most consistent evidence of benefit, while multimodal exercise also showed beneficial effects. However, the available evidence does not establish clear superiority of any single modality. Exercise selection should therefore consider the comparative evidence together with participant preferences, feasibility, accessibility, and the implementation setting.

**Systematic review registration:**

https://www.crd.york.ac.uk/PROSPERO/view/CRD420261359034, identifier (CRD420261359034).

## 1 Introduction

Body image is a multidimensional aspect of psychological well-being that encompasses perceptions, cognitive evaluations, and emotional experiences related to one’s physical appearance (Cash and Pruzinsky, 2002; Merino et al., 2024). Body-image concerns are particularly prevalent among women, reflecting the influence of sociocultural pressures, media-based appearance ideals, and interpersonal comparison (Tiggemann, 2011; Grogan, 2016; Czubaj et al., 2025; Sadık and Yılmaz, 2025). Negative body image has been associated with depression, anxiety, low self-esteem, disordered eating, and poorer quality of life (Abdoli et al., 2025; Rodgers et al., 2023). It may also contribute to broader psychopathology and difficulties in interpersonal functioning (Granero et al., 2026; Dai et al., 2025). Identifying effective approaches to improve body image is therefore relevant to both psychological health and quality of life in women.

Body-image concerns can be addressed through psychological, educational, and behavior-change approaches, but exercise offers a complementary non-pharmacological strategy that directly engages individuals with their bodies through movement and physical experience. Reviews and empirical studies have generally reported favorable associations between physical activity and body image across different populations (Sabiston et al., 2019; Gualdi-Russo et al., 2022; Srismith et al., 2022). Among women, greater physical activity has been associated with higher body esteem, body appreciation, and body-related self-concept and with lower body dissatisfaction (Tylka and Wood-Barcalow, 2015; Zhang et al., 2024; Thøgersen-Ntoumani et al., 2022; Belza and Warms, 2004). This relationship is also relevant from an intervention perspective because body dissatisfaction may itself discourage participation in physical activity (More et al., 2019).

Exercise is not a uniform intervention, and different modalities may influence body image through different pathways. Aerobic exercise may affect emotional regulation, neurochemical activity, inflammation, and body composition (Maurer et al., 2020; Lee et al., 2025). Resistance training may strengthen perceived physical capability, although responses can be shaped by sociocultural attitudes toward muscularity in women (Martin Ginis et al., 2014; Marashi et al., 2025). Dance combines movement with esthetic expression and social interaction and may therefore provide psychosocial benefits beyond those associated with conventional exercise (Boing et al., 2023; Marschin and Herbert, 2021). Mind–body practices such as yoga emphasize interoceptive awareness, breath regulation, and focused movement and have been associated with lower body surveillance and self-objectification (Cox et al., 2019; Balciuniene et al., 2022). More broadly, exercise may improve self-efficacy and promote a more functional orientation toward the body (Homan and Tylka, 2014; Greenleaf and Rodriguez, 2021). Body-image flexibility and exercise motivation may further shape these responses (Leung et al., 2023; Panão and Carraça, 2020).

Despite this evidence, the comparative effects of different exercise modalities remain uncertain. Previous reviews have examined physical activity and body image across heterogeneous populations and study designs (Sabiston et al., 2019; Srismith et al., 2022; Gualdi-Russo et al., 2022), but they do not establish the relative effectiveness of different exercise modalities within a single comparative framework. This distinction matters because the relationship between exercise and body image is not uniformly positive and may depend on the type of activity, participants’ motives, and their prior body-related experiences (Thøgersen-Ntoumani et al., 2022; Jankauskiene et al., 2024). Consequently, evidence remains limited regarding which exercise approaches are most consistently associated with improvements in body image among women.

Network meta-analysis (NMA) provides a useful framework for addressing this question because it integrates direct and indirect evidence and permits several interventions to be compared within the same analysis, even when head-to-head trials are limited. To our knowledge, no previous study has systematically compared different exercise modalities for women’s body image using this approach. We therefore conducted a systematic review and frequentist network meta-analysis of randomized controlled trials to compare aerobic exercise, resistance training, dance, mind–body exercise, and multimodal exercise, while retaining behavioral intervention as a separate active comparator.

## 2 Methods

### 2.1 Protocol and registration

This systematic review and network meta-analysis was conducted and reported in accordance with the PRISMA 2020 guidelines and the PRISMA extension for network meta-analyses (PRISMA-NMA), covering study selection, data extraction, and result reporting. The systematic review was registered in PROSPERO (CRD420261359034).

### 2.2 Data sources and search strategy

PubMed, Embase, Web of Science, and the Cochrane Library were searched from database inception to May 2026. The search strategy was informed by the PICOS (Population, Intervention, Comparison, Outcome, Study Design) framework and combined controlled vocabulary with free-text terms covering four core concepts: exercise, female participants, body image, and randomized controlled trials. Exercise terms included both general descriptors (e.g., exercise and physical activity) and modality-specific terms such as aerobic exercise, resistance training, yoga, tai chi, Pilates, and qigong. Body image terms included body image, body satisfaction, body esteem, body appreciation, and body dissatisfaction. Database-specific syntax and field restrictions were adapted to each platform. Study-design terms were used in PubMed, Embase, and Web of Science but were not applied in the Cochrane Library because CENTRAL is specifically designed to index controlled trials. Supplementary searching was also conducted through citation searching and hand-searching the reference lists of relevant systematic reviews. The complete reproducible search strategies for all four databases are provided in Supplementary Appendix B1.

### 2.3 Inclusion and exclusion criteria

Inclusion criteria. Studies were eligible if they met all of the following criteria:

1. Participants were females of any age with no contraindications to exercise, and only studies enrolling exclusively female samples were included.
2. Eligible active interventions included structured exercise programs comprising a single modality or a combination of modalities, as well as behavioral or educational programs retained as a separate active comparator node. Exercise programs were required to report the exercise type, total duration, weekly frequency, and session length. Only interventions lasting at least 4 weeks were eligible.
3. Eligible comparators included no intervention, waitlist, usual care, regular physical education, or other control conditions that did not receive the study-specific intervention under evaluation.
4. Body image was assessed using at least one validated and psychometrically sound instrument capturing constructs such as body satisfaction, body appreciation, social physique anxiety, or body esteem. Studies were required to report the pre- and post-intervention means and standard deviations for both groups, or data that could be converted into effect sizes.
5. The study was an original, peer-reviewed randomized controlled trial published in English.

Exclusion criteria. Studies were excluded if:

1. The study enrolled a male-only or mixed-sex sample.
2. The exercise intervention could not be isolated from co-administered psychological, dietary, or pharmacological components.
3. The publication was a qualitative study, review, or conference abstract.
4. The study used a non-interventional design, such as a cross-sectional, case–control, or cohort study.
5. The full text was not accessible, or key data required for effect-size calculation were unavailable.

### 2.4 Study selection

All retrieved records were imported into EndNote X9 and de-duplicated using automatic and manual checks. Two reviewers independently screened titles and abstracts and then assessed the full texts of potentially eligible reports. Disagreements were resolved through discussion, with a third reviewer consulted when necessary. Inter-reviewer agreement was assessed using Cohen’s kappa at both screening stages.

### 2.5 Data extraction

A standardized data extraction form was developed and piloted prior to use. Two authors (JLL and YHS) independently extracted the following information from each included study: (1) study characteristics, including first author, publication year, and country; (2) participant characteristics, including sample size per group and age; (3) intervention details, including exercise modality, intervention duration, weekly frequency, and session length; (4) comparator details; (5) outcome measures, including assessment instruments and pre- and post-intervention means and standard deviations for each group; and (6) methodological quality indicators. Disagreements were resolved by consensus.

### 2.6 Classification of exercise interventions

To construct a clinically interpretable network, we organized the interventions into six active nodes and one reference node. Exercise-based interventions were classified by their primary mode of movement or dominant physiological characteristic, informed by previous reviews of exercise and body image research (Zhang et al., 2024; Sabiston et al., 2019). Each intervention was assigned to a single node using a hierarchical rule based on the label reported in the original study (e.g., dance or yoga), the dominant physiological stimulus (aerobic, resistance, or mind–body), and the program structure (single or combined modality). Behavioral intervention was retained as a separate active comparator, while usual care served as the reference node. The exercise modalities were analyzed as distinct nodes rather than combined into a single intervention category. Their inclusion within the same network was justified by the use of a common target outcome, body image, in comparable female populations, with the evidence linked through a common reference structure. We also examined the transitivity assumption by considering potential effect modifiers, including participant age, intervention duration, and control type. This framework allowed clinically related but distinct interventions to be compared without assuming clinical equivalence.

Aerobic exercise: continuous, rhythmic activities involving large muscle groups that aim to improve cardiorespiratory function (e.g., walking, jogging, cycling, elliptical training).

Resistance training: activities that enhance muscular strength, endurance, or power through external resistance (e.g., free weights, resistance bands, machine-based or body-weight training, aquatic resistance training).

Dance: interventions in which structured or choreographed movement is the primary activity (e.g., aerobic dance, Zumba, ballet, creative dance).

Mind–body exercise: practices combining physical movement with mental focus, breath regulation, or meditation (e.g., yoga, tai chi, qigong, Pilates).

Multimodal exercise: structured programs combining two or more exercise modes without a clearly dominant component.

Behavioral intervention: psychological, educational, or behavior-change programs that were not defined by a specific exercise modality (e.g., health education, cognitive-behavioral programs, or physical-activity promotion programs). Behavioral intervention was treated as a separate active comparator rather than as an exercise modality because these programs were defined primarily by psychological, educational, or behavior-change strategies rather than by a specific mode of exercise. Keeping this node separate allowed behavioral approaches to be compared with exercise-based interventions and usual care within the same network.

Usual care: the reference node comprising no-intervention, waitlist, regular physical education, and other control conditions that did not receive the study-specific intervention under evaluation. These comparators were grouped because none involved the study-specific structured exercise intervention being evaluated and they provided a common reference that preserved connectivity across the treatment network. This grouping was operational rather than an assumption of clinical equivalence; in particular, regular physical education may differ meaningfully from passive controls in the amount of physical activity received. We therefore examined the influence of this decision in a sensitivity analysis excluding trials that used regular physical education as the control condition.

### 2.7 Risk of bias assessment

Two reviewers independently assessed the risk of bias in each included RCT using the RoB 2 tool. Risk of bias was evaluated across five domains: (1) bias arising from the randomization process; (2) bias due to deviations from intended interventions; (3) bias due to missing outcome data; (4) bias in measurement of the outcome; and (5) bias in selection of the reported result. Each domain was rated as low risk, some concerns, or high risk, and an overall risk-of-bias judgment was then derived for each study. Disagreements between reviewers were resolved through discussion, with a third reviewer consulted when necessary.

### 2.8 Statistical analysis

All statistical analyses were performed in R version 4.5. Because body image was measured using different validated instruments, treatment effects were expressed as SMDs with 95% CIs. Statistical heterogeneity was assessed using I^2^ and the between-study variance (τ^2^). I^2^ values of 50–75% were considered to indicate moderate heterogeneity and values above 75% high heterogeneity.

A frequentist random-effects network meta-analysis was conducted using the netmeta package (Balduzzi et al., 2023), with the DerSimonian–Laird estimator used for τ^2^. The network plot represented the available direct comparisons, with node size proportional to the number of participants and edge thickness proportional to the number of contributing studies. Pooled SMDs and 95% CIs were estimated for all treatment comparisons. For the multi-arm trial, the correlation arising from the shared control group was handled using the standard approach implemented in netmeta.

Agreement between direct and indirect evidence was assessed globally using the design-by-treatment interaction model and locally using node splitting. A *p* value greater than 0.05 was interpreted as no evidence of important inconsistency. Transitivity was examined by comparing potential effect modifiers across treatment comparisons, including participant age, intervention duration, and control type. The exercise modalities were retained as separate treatment nodes because they addressed the same body-image outcome in comparable female populations and were connected through a common reference structure.

Treatments were ranked using P-scores (Rücker and Schwarzer, 2017; Salanti et al., 2011). P-scores range from 0 to 1, with higher values indicating a more favorable relative position within the network. Rankings were interpreted together with effect estimates and confidence intervals and were not treated as probabilities that an intervention was the single best treatment.

Small-study effects and publication bias were assessed using complementary network-level and pooled pairwise approaches. At the network level, a comparison-adjusted funnel plot was constructed and asymmetry was examined using Egger’s regression test (Chaimani et al., 2013; Egger et al., 1997). A conventional pairwise random-effects meta-analysis was also used to generate a funnel plot and conduct Egger’s test and trim-and-fill analysis (Duval and Tweedie, 2000). These assessments were interpreted cautiously because asymmetry tests have limited reliability when few studies contribute to individual comparisons (Sterne et al., 2011).

Robustness was examined in two sensitivity analyses. First, a conventional pairwise random-effects meta-analysis using the restricted maximum-likelihood estimator was combined with leave-one-out analysis (Supplementary Figure S2). Second, because the usual-care node included heterogeneous control conditions, the network meta-analysis was repeated after excluding trials in which the control group received regular physical education (Hajihosseini, 2015; Halliwell et al., 2018; Kahlin et al., 2015; Zhu and Wen, 2025). The restricted network was compared with the primary analysis. Statistical significance was set at α = 0.05.

## 3 Results

### 3.1 Trial selection

**Figure 1:**
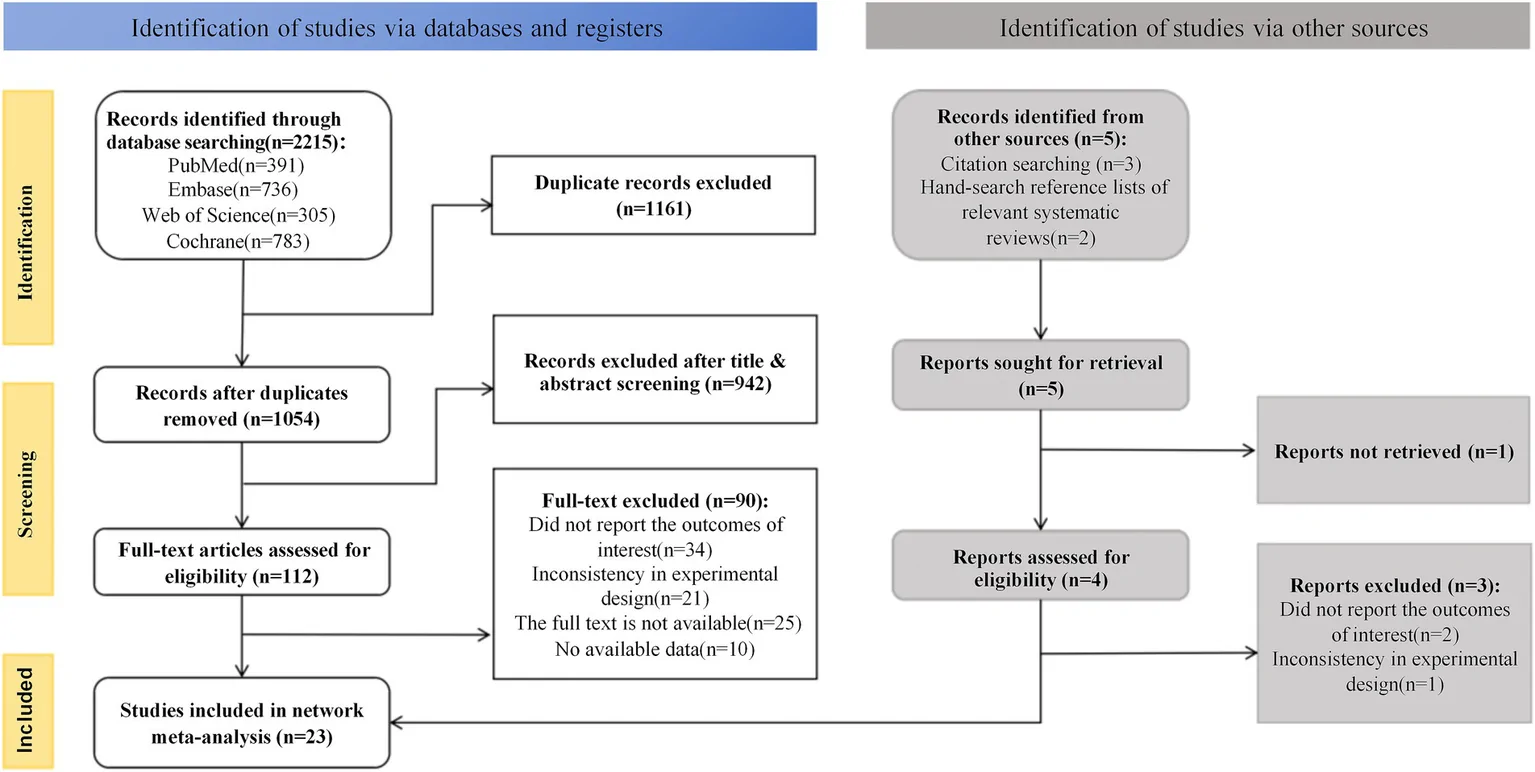
PRISMA flow diagram of study selection process.

A total of 2,215 records were identified from four electronic databases: 391 from PubMed, 783 from the Cochrane Library, 736 from Embase, and 305 from Web of Science. After removing 1,161 duplicates, 1,054 unique records remained for screening. Two reviewers (JLL and YHS) independently screened titles and abstracts and assessed potentially eligible full texts. Inter-reviewer agreement was substantial at both stages, with Cohen’s κ = 0.76 for title/abstract screening and Cohen’s κ = 0.83 for full-text assessment. During title and abstract screening, 942 records were excluded, leaving 112 for full-text assessment. At the full-text stage, 90 records were excluded according to the eligibility criteria (did not report outcomes of interest, *n* = 34; inconsistency in experimental design, *n* = 21; full text not available, *n* = 25; no available data, *n* = 10). In addition, five records were identified through supplementary searching. Of these, one report could not be retrieved, three were excluded after full-text assessment, and one met all eligibility criteria and was included in the final analysis. In total, 23 RCTs were included in the network meta-analysis (Figure 1). The complete database-specific search strategies are provided in Supplementary Appendix B1.

### 3.2 Characteristics of included studies

The 23 included RCTs were published between 2001 and 2025, were conducted across 13 countries, and enrolled a total of 1,914 female participants; their characteristics are summarized in Table 1. Participants ranged from preadolescent girls to older women.

**Table 1:** Characteristics of included randomized controlled trials.

No.	Study	Country	N (IG; CG)	Age (IG; CG)	Intervention (IG)			Intervention (CG)			Population	Outcomes
					Intervention content	Intervention time, frequency, period	Type	Intervention content	Intervention time, frequency, period	Type		
1	Alleva et al. (2020)	Netherlands	58;56	22.19 ± 2.42	Hatha yoga	10 weeks, once weekly for 60 min	Mind–Body	Usual Care	10 weeks	Usual Care	Young women	BAS-2
2	Annesi et al. (2011)	United States	63;51	42.6 ± 10.3	The Coach Approach	6 months, exercise 3 times/week + monthly counseling	Behavioral	Standard Fitness Practice	6 months	Usual Care	Middle-aged women	Body Areas Satisfaction
3	Baptista et al. (2012)	Brazil	40;40	49.5;49.1	Belly dance	16 weeks, twice weekly for 60 min	Dance	Usual Care	16 weeks	Usual Care	Middle-aged women	BDDE
4	Cowley et al. (2021)	United Kingdom	22;20	14.2 ± 1.1	home-based multi-component Physical activity	6 weeks, 3 × 30 min	Multimodal	Waitlist	6 weeks	Usual Care	Adolescent girls	BAS
5	Dittrich et al. (2008)	Austria	15;15	33.7 ± 12.5;32.1 ± 12.1	aerobic exercise + progressive muscle relaxation	6 weeks, twice weekly for 60 min	Aerobic	Usual Care	6 weeks	Usual Care	Young women	FKB-20
6	Duncan et al. (2009)	United Kingdom	17;17	10–11	circuit training	6 weeks, twice weekly	Multimodal	No extra exercise	6 weeks	Usual Care	Preadolescent girls	BES
7	Hajihosseini (2015)	Iran	20;21	12.9 ± 0.55;13 ± 0.63	fitness + traditional games	16 weeks, twice weekly for 30 min	Multimodal	Regular Physical Education	16 weeks	Usual Care	Adolescent girls	PSDQ
8	Halliwell et al. (2018)	United Kingdom	91;95	9.34 ± 0.69	Yoga	4-week yoga, once weekly, 40 min per session	Mind–body	Regular physical education classes	4-week	Usual Care	Preadolescent girls	BAS-2
9	Halliwell et al. (2019)	United Kingdom	22;22	20.21 ± 2.15	Yoga	4-week yoga intervention, 60 min/week, body image themes	Mind–body	Usual Care	4-week	Usual Care	Young women	BAS-2
10	Kahlin et al. (2015)	Sweden	55;39	17.2 ± 0.55;16.1 ± 0.34	Mixed physical activity (gym + aerobic)	6 months, ≥1 time/week self-selected	Multimodal	Usual school PE	6 months	Usual Care	Adolescent girls	CY-PSPP
11	Khalili et al. (2022)	Iran	31;31	64.2 ± 3.1;64.2 ± 2.6	Group walking	8 weeks, three times weekly for 30 min	Aerobic	Usual Care	8 weeks	Usual Care	Older women	SPA
12	Legrand (2014)	France	15;12	21.56 ± 3.18;20.59 ± 2.18	aerobic exercise	7 weeks, twice weekly for 60 min	Aerobic	Waitlist	7 weeks	Usual Care	Young women	ISP-25
13	Legrand and Crombez-Bequet (2021)	France	11;10	33.4 ± 3.5	walking/running + strength	6 weeks, twice weekly for 35–40 min	Multimodal	No Intervention	6 weeks	Usual Care	Young women	ISP-25
14	Li et al. (2002)	United States	40;32	72.8 ± 4.7;72.7 ± 5.7	Tai Chi	6 months, twice weekly for 60 min	Mind–Body	Waitlist	6 months	Usual Care	Older women	PSPP
15	Lindwall and Lindgren (2005)	Sweden	27;35	15.2 ± 1.7;15.4 ± 1.0	various self-selected activities	6 months, twice weekly 45 min exercise + 15 min discussion	Multimodal	Waitlist	6 months	Usual Care	Adolescent girls	PSPP
16	Martínez-Rodríguez et al. (2021)	Spain	17;17	69.6 ± 5;67.7 ± 3.6	aquatic resistance interval training	14 weeks, three times weekly for 60 min	Resistance	nutrition education only	14 weeks	Usual Care	Older women	BSQ
17	Megakli et al. (2015)	Greece	37;35	32.7 ± 7.27;32.22 ± 8.84	aerobic + resistance combination	12 weeks, three times weekly for 60 min	Multimodal	Usual Care	12 weeks	Usual Care	Young women	PSPP
18	Müller-Pinget et al. (2018)	Switzerland	27;19	46.9 ± 10.2	Dance therapy	16 weeks, once weekly for 120 min	Dance	Usual Care	16 weeks	Usual Care	Middle-aged women	BE
19	Musanti (2012)	United States	10;12	50.5 ± 7.5	Aerobic	12 weeks, 3–5 times/week	Aerobic	Usual Care	12 weeks	Usual Care	Middle-aged women	PSPP
19	Musanti (2012)	United States	9;12	50.5 ± 7.5	Resistance	12 weeks, 3–5 times/week	Resistance	Usual Care	12 weeks	Usual Care	Middle-aged women	PSPP
19	Musanti (2012)	United States	11;12	50.5 ± 7.5	Aerobic + resistance	12 weeks, 3–5 times/week	Multimodal	Usual Care	12 weeks	Usual Care	Middle-aged women	PSPP
20	Stice et al. (2013)	United States	198;200	17–20;17–20	Healthy Weight education (promote physical activity)	4 weeks, once weekly 60 min group	Behavioral	educational brochure	4 weeks	Usual Care	Young women	BDS
21	Yìğìter (2014)	Turkey	40;40	21.52 ± 1.43;22.15 ± 1.67	regular exercise (mixed aerobic/ball games)	12 weeks, three times weekly for 60 min	Multimodal	Usual Care	12 weeks	Usual Care	Young women	SE
22	Zabinski et al. (2001)	United States	80;97	24 ± 1.95	GRAD health course (promote physical activity)	15 weeks, weekly 1 h lecture + 1.5 h lab	Behavioral	general health lecture	15 weeks	Usual Care	Young women	BDS
23	Zhu and Wen (2025)	China	21;21	18–25;18–25	basketball	10 weeks, twice weekly for 90 min	Multimodal	standard PE	10 weeks	Usual Care	Young women	BAS-2

IG, Intervention Group; CG, Control Group; BAS-2, Body Appreciation Scale-2; BAS, Body Appreciation Scale; BDDE, Body Dysmorphic Disorder Examination; BES, Body Esteem Scale; PSDQ, Physical Self-Description Questionnaire; FKB-20, Fragebogen zum Körperbild-20 (Body Image Questionnaire-20); CY-PSPP, Children and Youth Physical Self-Perception Profile; PSPP, Physical Self-Perception Profile; SPA, Social Physique Anxiety Scale; ISP-25, French version of the Physical Self-Perception Profile (25-item); BSQ, Body Shape Questionnaire; BE, Body Evaluation subscale; BDS, Body Dissatisfaction Scale; SE, Rosenberg Self-Esteem Scale; Musanti (2012) was a multi-arm parallel trial; all three exercise arms shared one identical usual-care control group (*n* = 12); Usual Care encompasses no-intervention, waitlist, regular physical education, and other control conditions that did not receive the study-specific intervention under evaluation.

Intervention duration ranged from 4 weeks to 6 months, while weekly frequency and session length varied substantially across programs. Body image was assessed using a range of validated instruments, including the Body Appreciation Scale (BAS), Body Appreciation Scale-2 (BAS-2), Physical Self-Perception Profile (PSPP), Children and Youth Physical Self-Perception Profile (CY-PSPP), Body Shape Questionnaire (BSQ), Body Esteem Scale (BES), Body Dissatisfaction Scale (BDS), Body Dysmorphic Disorder Examination (BDDE), Social Physique Anxiety Scale (SPA), and other body image related measures.

The interventions comprised six active categories, together with usual care as the reference: aerobic exercise (4 studies), dance (2 studies), resistance training (2 studies), mind–body exercise (4 studies), multimodal exercise (10 studies), and behavioral intervention (3 studies). One multi-arm trial (Musanti, 2012) contributed three active intervention arms, including aerobic, resistance, and multimodal exercise, which shared a common usual-care control group. The correlation induced by this shared control group was handled using the standard netmeta approach to avoid unit-of-analysis errors.

### 3.3 Risk of bias

**Figure 2:**
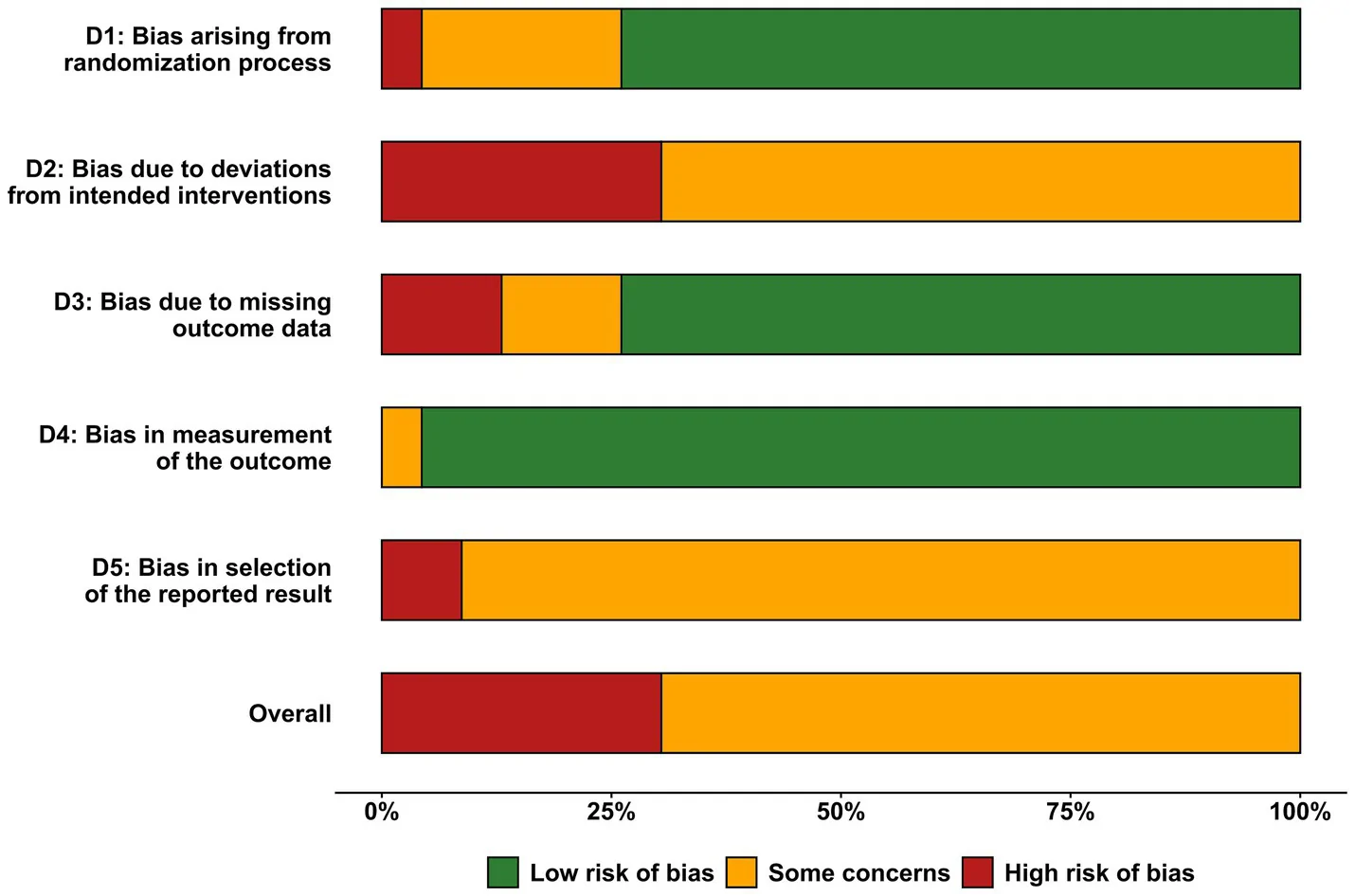
Risk of bias assessment of included studies using the Cochrane RoB 2 tool.

The RoB 2 assessments are summarized in Figure 2, with study-level judgments presented in the Supplementary Figure S1. Sixteen studies were judged as having some concerns and seven as having high overall risk of bias; no study was rated as low overall risk. Domain 2, concerning deviations from intended interventions, was the most frequent source of concern. By contrast, outcome measurement (Domain 4) was judged as low risk in nearly all studies. High-risk judgments in individual trials also arose from concerns related to randomization, missing outcome data, and selection of the reported result.

### 3.4 Network meta-analysis

#### 3.4.1 Heterogeneity and model selection

Global heterogeneity in the network was moderate (I^2^ = 59%, τ^2^ = 0.087, τ = 0.29). Accordingly, the random-effects model specified for the primary analysis was retained. To further assess robustness, a conventional pairwise random-effects meta-analysis pooling all included studies, together with a leave-one-out sensitivity analysis, was performed; both are presented in Supplementary Figure S2. The pooled estimate (SMD = 0.33, 95% CI: 0.17–0.49; I^2^ = 62.2%) remained stable when any single study was omitted, indicating that the pooled effect was not driven by any one study.

#### 3.4.2 Evidence network

**Figure 3:**
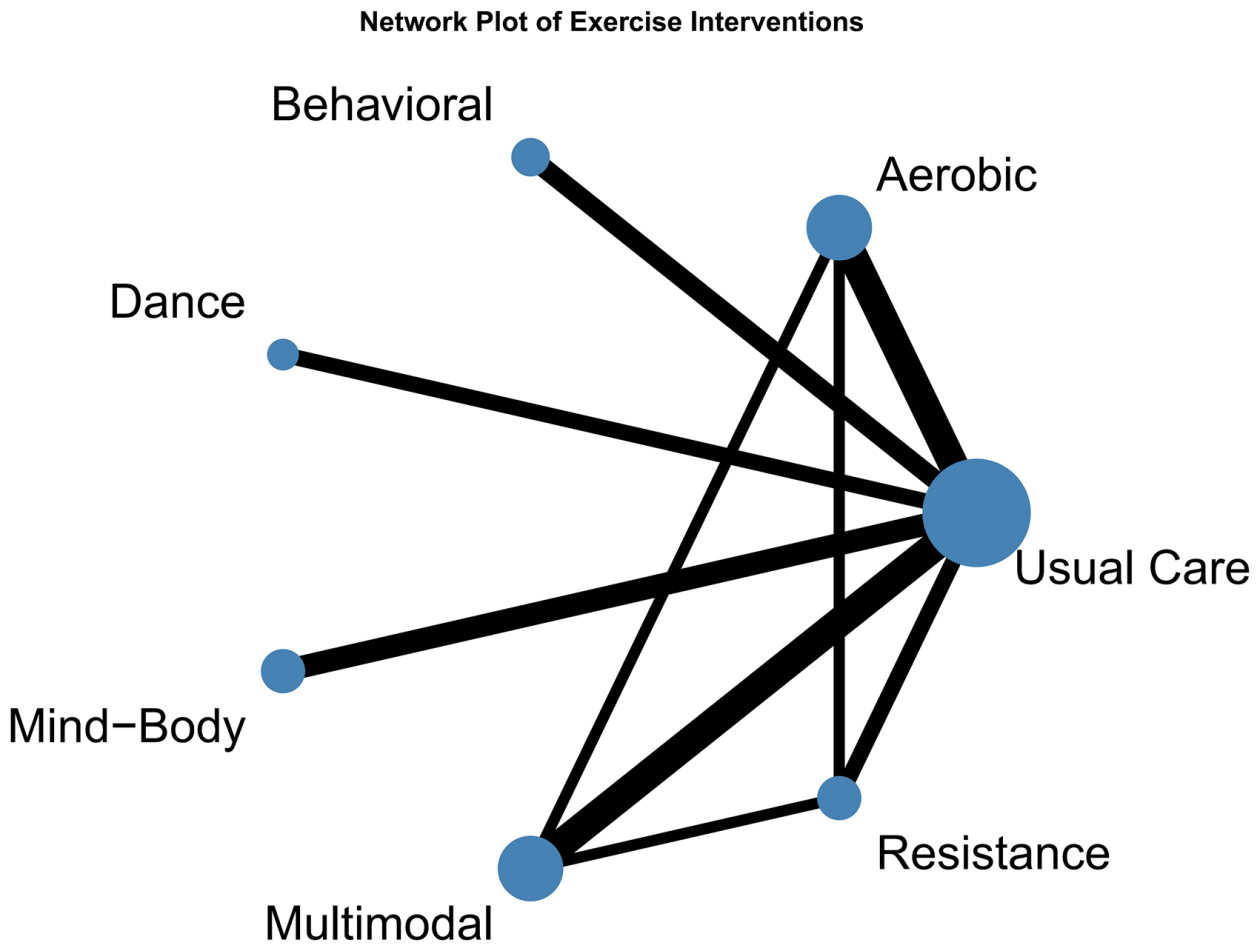
Evidence network plot for the network meta-analysis.

The evidence network is shown in Figure 3. Usual care, serving as the reference node, was directly connected to all six active intervention nodes, and direct head-to-head comparisons were available among aerobic exercise, resistance training, and multimodal exercise. This connected network structure supported the estimation of indirect comparisons across all nodes.

#### 3.4.3 Consistency assessment

Node-splitting analysis revealed no significant inconsistency between direct and indirect evidence in any closed loop (all *p* > 0.05), and the global design-by-treatment interaction test likewise indicated no important inconsistency. A consistency model was therefore used for all subsequent analyses.

#### 3.4.4 Effects of exercise interventions on body image

**Figure 4:**
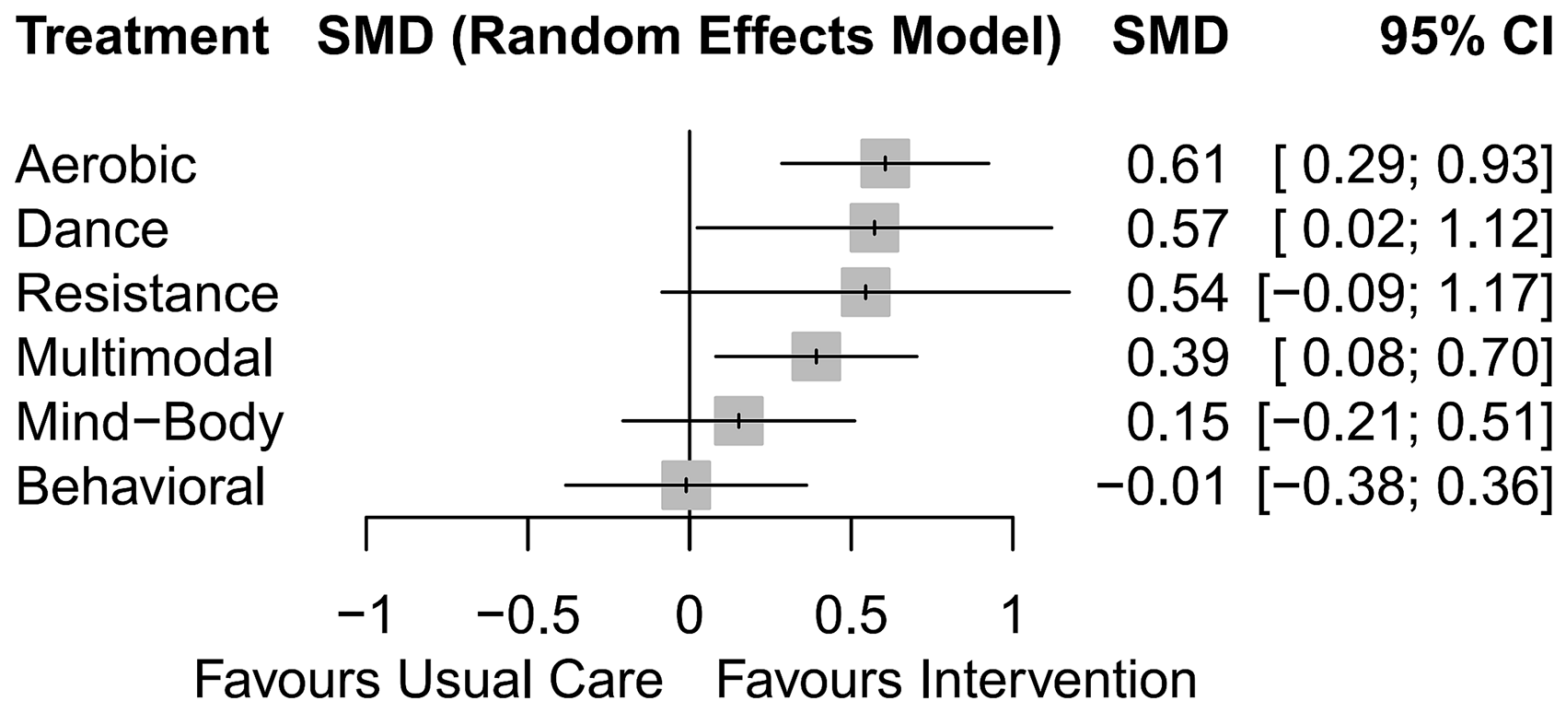
Forest plot of network meta-analysis results.

**Figure 5:**
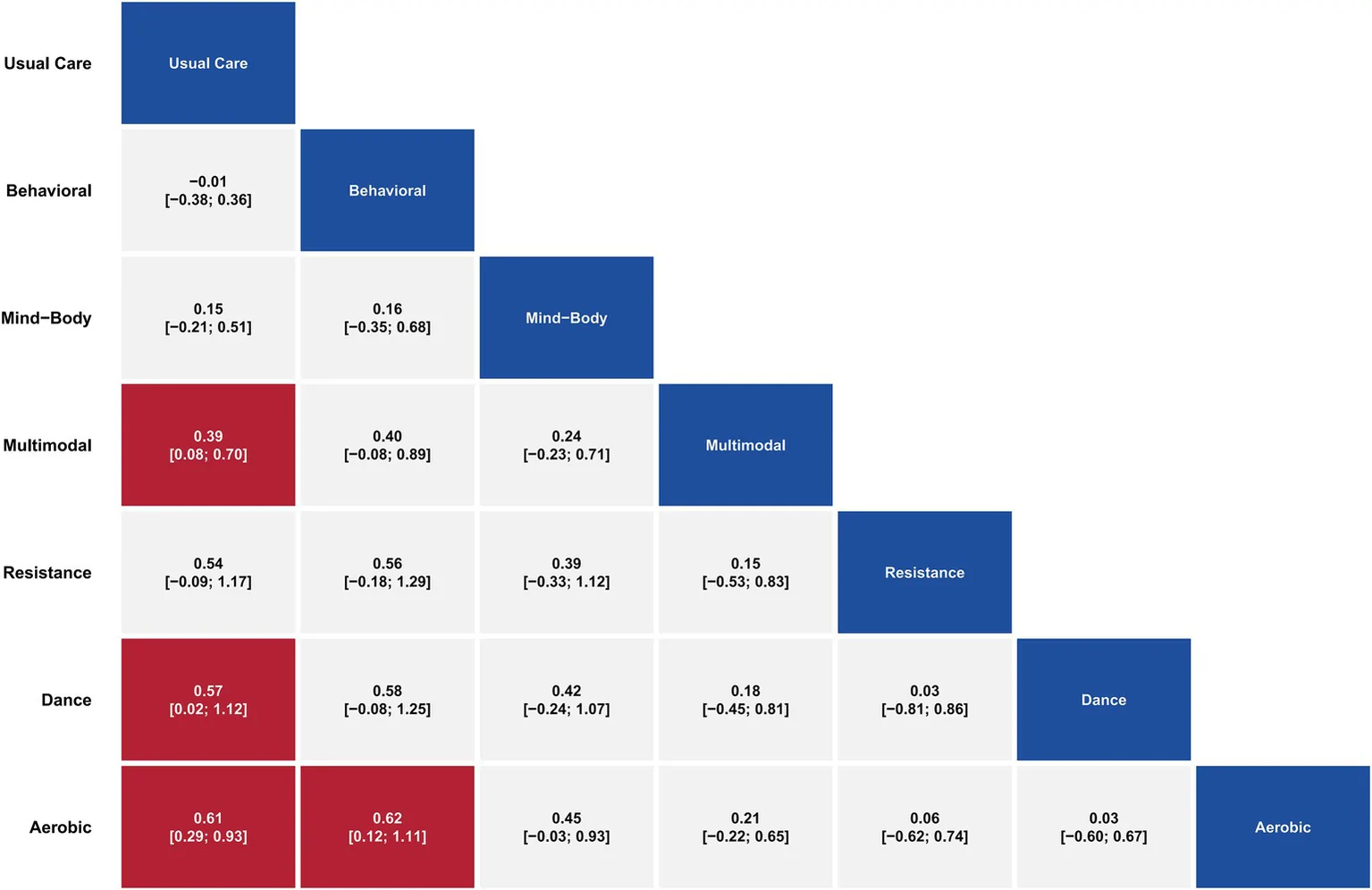
League table of pairwise comparisons among all interventions (random-effects network meta-analysis). Values are SMDs (95% CIs) for the intervention defining the row compared with the intervention defining the column; positive values favor the row intervention. Red cells indicate statistically significant comparisons (95% CI excludes zero); gray cells indicate non-significant comparisons. Only the lower triangle is presented, as the upper triangle would contain reciprocal, redundant comparisons.

The NMA results are presented in Figure 4 and summarized in the league table in Figure 5. Compared with usual care, three modalities showed statistically significant improvements in body image. Aerobic exercise showed the largest effect with a relatively precise estimate (SMD = 0.61, 95% CI: 0.29 to 0.93), followed by dance (SMD = 0.57, 95% CI: 0.02 to 1.12) and multimodal exercise (SMD = 0.39, 95% CI: 0.08 to 0.70). Resistance training showed a moderate but non-significant effect (SMD = 0.54, 95% CI: −0.09 to 1.17), while mind–body exercise (SMD = 0.15, 95% CI: −0.21 to 0.51) and behavioral intervention (SMD = −0.01, 95% CI: −0.38 to 0.36) did not differ significantly from usual care. Pairwise comparisons among the active interventions are presented in Figure 5. Aerobic exercise was significantly more effective than behavioral intervention (SMD = 0.62, 95% CI: 0.12 to 1.11). No other comparisons among the active interventions reached statistical significance. In particular, aerobic exercise was not significantly more effective than dance, resistance training, multimodal exercise, or mind–body exercise (aerobic vs. Mind–body: SMD = 0.45, 95% CI: −0.03 to 0.93), and multimodal exercise did not differ significantly from behavioral intervention (SMD = 0.40, 95% CI: −0.08 to 0.89). Given the substantial overlap in confidence intervals and the absence of statistically significant differences among most active-treatment comparisons, the available evidence does not establish clear superiority of any single exercise modality.

#### 3.4.5 Intervention ranking

**Figure 6:**
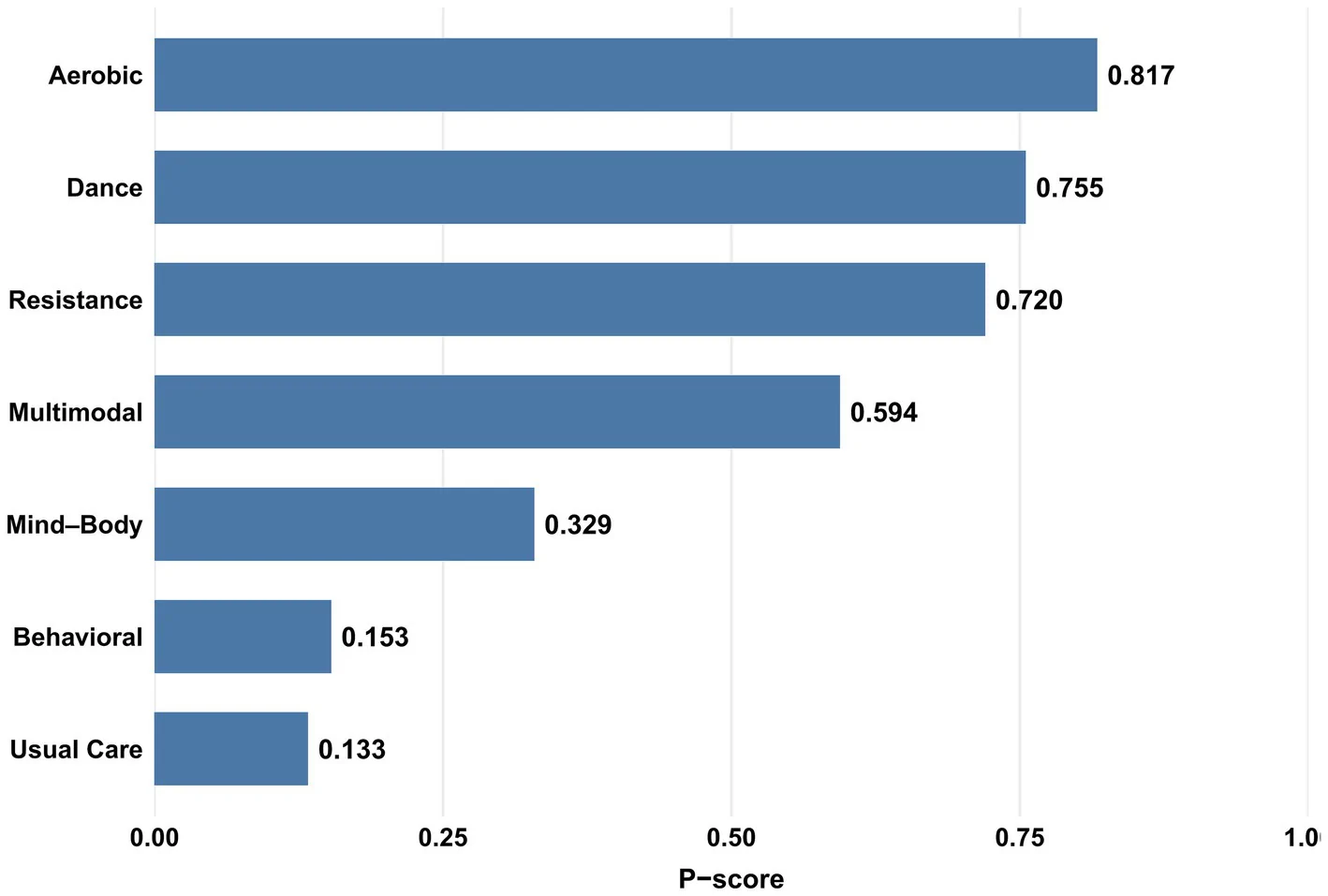
Intervention rankings based on P-scores.

P-scores were used to summarize the relative ordering of interventions within the network (Table 2; Figure 6). Aerobic exercise had the highest P-score (0.817), followed by dance (0.755), resistance training (0.720), and multimodal exercise (0.594); lower values were observed for mind–body exercise (0.329), behavioral intervention (0.153), and usual care (0.133). These rankings should not be interpreted as evidence that the highest-ranked intervention is clearly superior. Confidence intervals for the leading interventions overlapped substantially, and differences in P-scores should therefore be considered alongside the corresponding comparative effect estimates. Moreover, among the active interventions, only the comparison between aerobic exercise and behavioral intervention was statistically significant. Thus, P-scores describe the relative ordering of interventions within the network but do not establish clear superiority among the leading exercise modalities.

**Table 2:** Intervention ranking based on P-scores from the network meta-analysis (random-effects model).

Rank	Intervention	P-score
1	Aerobic	0.817
2	Dance	0.755
3	Resistance	0.720
4	Multimodal	0.594
5	Mind–Body	0.329
6	Behavioral	0.153
7	Usual Care	0.133

### 3.5 Sensitivity analysis

To examine whether inclusion of trials using regular physical education as the control condition influenced the findings, we repeated the network meta-analysis after excluding four such trials (Hajihosseini, 2015; Halliwell et al., 2018; Kahlin et al., 2015; Zhu and Wen, 2025). The overall pattern remained broadly similar (Supplementary Figure S4; Supplementary Table S1). Aerobic exercise (SMD = 0.60, 95% CI: 0.35 to 0.86) and dance (SMD = 0.58, 95% CI: 0.15 to 1.00) remained significantly superior to usual care, and mind–body exercise became statistically significant (SMD = 0.36, 95% CI: 0.04 to 0.68). In the restricted network, the relative ordering was aerobic exercise (P-score = 0.837), dance (0.787), resistance training (0.704), mind–body exercise (0.558), and multimodal exercise (0.370).

The relative ordering of mind–body and multimodal exercise was reversed after removal of the trials using regular physical-education controls, suggesting that the ranking of these interventions was sensitive to control-group composition. Because confidence intervals overlapped substantially, the lower- and middle-ranked interventions should be interpreted cautiously. Aerobic exercise and dance remained significantly superior to usual care in the restricted analysis, indicating that their favorable effects were not dependent on inclusion of the regular physical-education control trials. Heterogeneity was also substantially lower in the restricted network (I^2^ = 28.9%) than in the primary analysis (I^2^ = 59%), suggesting that variation in control conditions contributed meaningfully to the overall heterogeneity.

### 3.6 Publication bias

**Figure 7:**
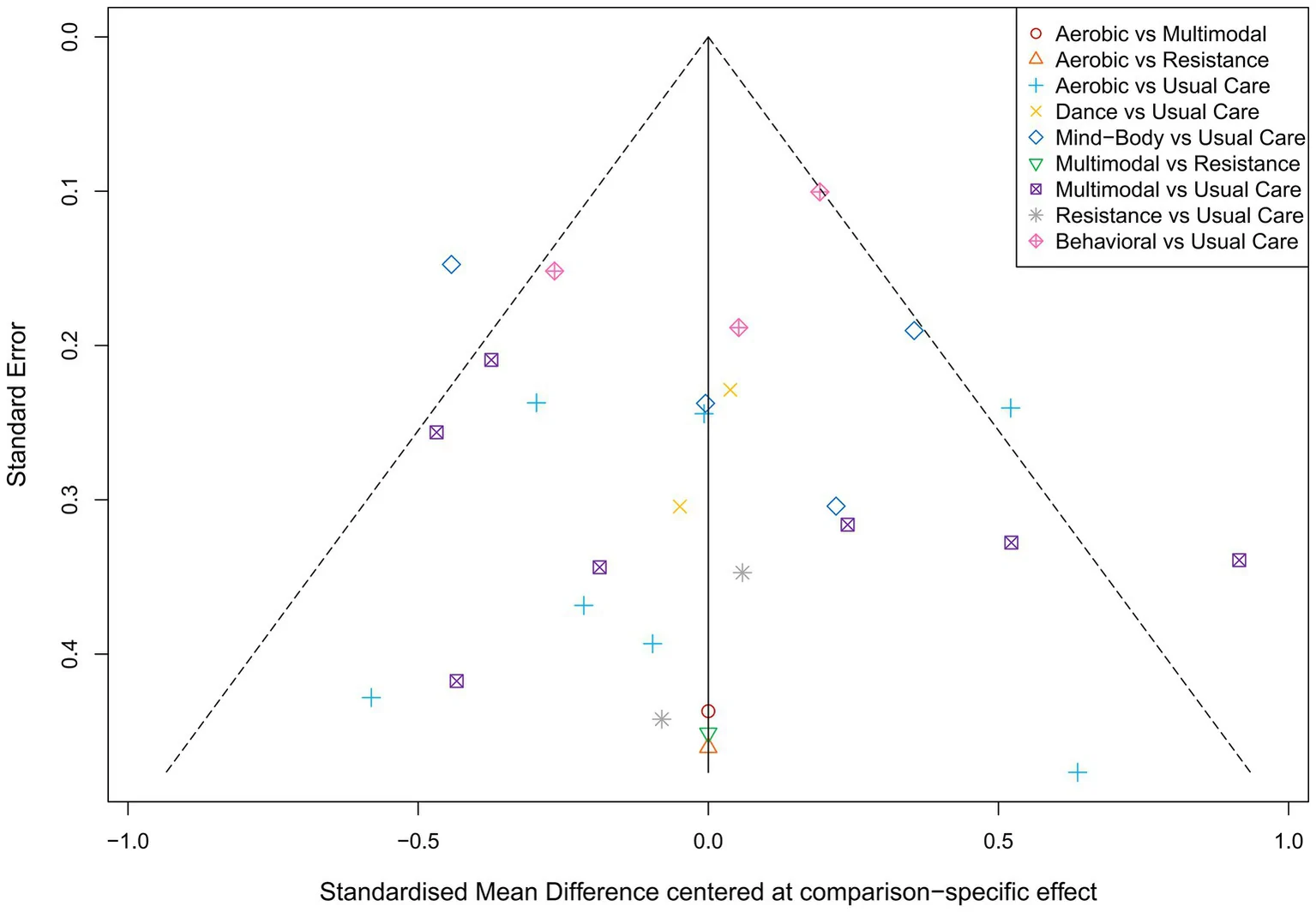
Comparison-adjusted funnel plot for the assessment of small-study effects across the network. Each point represents a study contributing to a specific comparison, with effect sizes centered at the comparison-specific pooled effect. Treatments were ordered from the assumed most to least effective for construction of the plot.

Publication-bias assessments produced different results across the two analytical approaches. The comparison-adjusted network funnel plot showed no clear asymmetry, and the network-level Egger’s test was not significant (*p* = 0.887). In contrast, the pooled pairwise Egger’s test indicated asymmetry (*p* = 0.022). Trim-and-fill imputed 11 potentially missing studies and attenuated the pooled estimate from SMD = 0.33 (95% CI: 0.17 to 0.49) to SMD = 0.06 (95% CI: −0.12 to 0.25). Given these discordant findings, publication bias could not be excluded (Figure 7).

## 4 Discussion

This systematic review and network meta-analysis synthesized 23 randomized controlled trials involving 1,914 female participants. Aerobic exercise, dance, and multimodal exercise showed significant benefits relative to usual care, whereas the estimate for resistance training favored the intervention but did not reach statistical significance. Although aerobic exercise had the highest P-score, the comparative estimates for the leading exercise modalities were not clearly separated: their confidence intervals overlapped substantially, and no significant advantage of aerobic exercise over dance, resistance training, multimodal exercise, or mind–body exercise was observed. The ranking should therefore be read as a description of relative position within the network rather than as a hierarchy of clinically established superiority.

Moderate heterogeneity was present across the network (I^2^ = 59%), reflecting variation in both the interventions and the populations studied. Intervention duration, weekly frequency, and session length differed across trials, and exercise intensity was also likely to vary. Participant characteristics also differed across studies. A further source of heterogeneity was the measurement of body image itself. The included instruments assessed related but distinct constructs, including body appreciation, body esteem, body dissatisfaction, and social physique anxiety. Standardization allowed these outcomes to be analyzed on a common scale, but it does not remove differences in the underlying constructs or their psychometric properties.

Control-group composition may also have contributed to this variability. After trials using regular physical education as an active control were excluded, network heterogeneity fell from I^2^ = 59 to 28.9%. Aerobic exercise and dance nevertheless remained significantly superior to usual care. Thus, the direction of these findings was relatively stable, although some effect estimates were sensitive to the composition of the control group. The pooled estimates should accordingly be understood as average effects across studies that differed in their populations, intervention protocols, and control conditions.

The sensitivity analysis also changed the relative ordering of mind–body and multimodal exercise after the regular physical-education control trials were removed. The evidence base was also unevenly distributed across intervention nodes, with some modalities represented by only a small number of trials, reducing the precision of their estimates and potentially contributing to instability in the comparative rankings. These considerations are important when interpreting the treatment rankings. Small differences between intervention nodes, particularly when their confidence intervals overlap, provide limited grounds for distinguishing their relative effectiveness and should not be taken as evidence of stable or clinically important differences between modalities.

The favorable effects of aerobic exercise may partly reflect its influence on mood regulation and broader neurobiological processes. Aerobic exercise has been associated with changes in neurochemical activity, inflammatory processes, and emotional regulation, which may in turn contribute to more positive body-related evaluations (Heijnen et al., 2016). From a behavioral perspective, common aerobic activities such as walking, cycling, and jogging can be incorporated into regular activity routines, which may support sustained participation and gradual improvements in body image (Nakagawa et al., 2020). These findings are consistent with previous research reporting favorable effects of aerobic exercise on body image-related outcomes (Salci and Martin Ginis, 2017). Aerobic exercise may also produce changes in body composition, including reductions in body fat, although such changes should not be assumed to translate directly into improvements in body image (Lafontant et al., 2025). However, responses may differ among individuals with severe body image concerns or eating disorders, and excessive exercise may be associated with compulsive exercise tendencies (Lichtenstein et al., 2017).

Dance also showed a significant benefit relative to usual care. Unlike forms of exercise that place greater emphasis on performance or weight-related outcomes, dance combines rhythmic movement, expressive action, and social interaction. These features may reduce appearance-based self-evaluation and encourage a more functional and self-reflective relationship with the body (Yu et al., 2025). Dancing may also shift attention from appearance toward movement and expression, potentially reducing body surveillance and weight-related concerns (Liu and Vorawattanachai, 2026). Group-based dance programs may further provide social support and reduce appearance-related social comparison, both of which have been linked to lower body dissatisfaction in women (Patterson et al., 2022; Liu et al., 2025). Evidence from clinical populations, including breast cancer survivors, has likewise shown improvements in body self-perception and functional health following dance interventions (Boing et al., 2023).

Resistance training showed a moderate but imprecise effect that did not reach statistical significance. The limited number of available trials reduces the precision of this estimate, and the non-significant result should not be interpreted as evidence of no effect. One possible explanation for variation in response concerns sociocultural norms that prioritize leanness over muscularity in idealized female body standards. In some women, increases in muscle mass or perceived bulk may conflict with internalized appearance ideals and attenuate improvements in body image (Foster et al., 2015). Framing resistance training around functional outcomes such as strength, independence, and physical capability may therefore be more conducive to improvements in body satisfaction (Marashi et al., 2025).

Multimodal exercise also showed a significant benefit relative to usual care. Programs that combine different exercise modes may provide complementary physical and psychological benefits (Malliou et al., 2025). Aerobic components may support affective regulation, while resistance components may enhance perceived strength and physical capability. This combination may be particularly relevant for women whose body image concerns extend beyond appearance to perceptions of physical function (Kamimura et al., 2014; Lacroix et al., 2023). Greater variety may also reduce monotony and support engagement over time (Fairchild et al., 2024). Although the point estimate for multimodal exercise was lower than those for aerobic exercise and dance, the confidence intervals overlapped substantially, providing little evidence of a clear difference between these modalities.

Mind–body exercises such as yoga, tai chi, and qigong showed a small, non-significant effect. This finding should not be interpreted as evidence of ineffectiveness. These interventions may improve body image through greater interoceptive awareness, reduced self-objectification, and increased acceptance of bodily experience. Such psychological changes may develop gradually and could require longer intervention periods than those used in some of the included RCTs (Gutiérrez-Cabrero and González-García, 2025). Differences in outcome instruments may also have reduced sensitivity to domain-specific changes. Longitudinal and observational studies have reported more consistent improvements in body appreciation with sustained practice, suggesting that short-term RCTs may underestimate some of the potential benefits of mind–body exercise (Jankauskiene et al., 2024). These temporal differences may partly account for the smaller point estimate observed for mind–body exercise relative to aerobic exercise, although the between-modality difference was not statistically significant.

Behavioral intervention had a negligible pooled effect on body image. This pattern may indicate that interventions involving direct physical engagement influence body image through pathways that differ from those targeted primarily by cognitive or educational approaches (Morano et al., 2020). Psychoeducational and cognitive-behavioral approaches can address body-related beliefs and exercise motivation directly, while research on women’s physical-activity experiences also suggests that motives and the meaning attached to exercise may shape engagement with physical activity (O'Dougherty et al., 2010; More et al., 2022). Interpretation of this node is further complicated by substantial clinical heterogeneity across programs that differed in duration, intensity, and intervention content. Differences in treatment intensity and theoretical orientation may therefore have contributed to the pooled estimate and limit conclusions about behavioral interventions as a single category.

The two approaches used to assess small-study effects produced different results. The comparison-adjusted network analysis did not indicate clear asymmetry, whereas asymmetry was detected in the pooled pairwise analysis. Trim-and-fill further attenuated the pooled pairwise effect, with the adjusted confidence interval crossing zero. These findings do not support a firm conclusion either for or against publication bias. The discrepancy may partly reflect differences in the structure of the two analyses. The pooled pairwise analysis combines intervention-control comparisons with different underlying effects, whereas the comparison-adjusted network approach accounts for comparison-specific effects before assessing asymmetry. Because relatively few trials contributed to individual comparisons, neither analysis provides a definitive assessment. Publication bias therefore remains a source of uncertainty, and some overestimation of intervention effects cannot be excluded.

## 5 Strengths and limitations

This study has several strengths. To our knowledge, it is the first network meta-analysis to systematically compare the relative effects of different exercise modalities on body image in women. By integrating direct and indirect evidence within a unified framework, the analysis allows multiple modalities to be compared simultaneously, extending beyond conventional pairwise comparisons. The review was conducted and reported in accordance with PRISMA-NMA guidelines, and risk of bias was systematically assessed using the Cochrane RoB 2 tool. The wide age range of the included female samples also broadens the population scope of the review.

Several limitations should be acknowledged. First, blinding of participants and instructors is often impractical in exercise trials, which may increase the potential for deviations from intended interventions. Consistent with the RoB 2 assessment, most studies were judged as having “some concerns,” and seven were rated at high overall risk of bias. Second, moderate heterogeneity was observed across the network (I^2^ = 59%), likely reflecting differences in intervention duration, frequency and intensity, participant characteristics, outcome instruments, and control conditions. Although SMDs placed the different body image measures on a common metric, they cannot eliminate clinical differences between the constructs captured by these instruments. The pooled estimates should therefore be interpreted as average effects across heterogeneous study settings rather than as effects expected uniformly across all populations and intervention protocols. Third, the evidence base for resistance training and behavioral intervention was limited, resulting in wide confidence intervals and reduced precision of the effect estimates. Fourth, publication bias could not be excluded. The comparison-adjusted network analysis showed no evidence of asymmetry, whereas asymmetry was detected in the pooled pairwise analysis and trim-and-fill substantially attenuated the pooled effect. Because few trials contributed to most comparisons, neither approach provides a definitive assessment. The magnitude of some intervention effects may therefore be overestimated. Fifth, restricting eligibility to English-language publications may have resulted in the omission of relevant trials reported in other languages. This restriction may have reduced the completeness of the evidence base and could have affected the pooled estimates if study availability or reported effects differed systematically by publication language. It may also limit the generalizability of the findings across cultural settings, which is relevant because body image perceptions are partly shaped by sociocultural context. The direction and magnitude of any resulting language bias cannot be determined from the available evidence. Sixth, the control conditions were not fully homogeneous, as some studies used regular physical education rather than passive controls. Although aerobic exercise and dance remained significantly superior to usual care in the sensitivity analysis, heterogeneity in the restricted network was markedly lower (I^2^ = 28.9%), suggesting that differences in control-group composition contributed to the heterogeneity observed in the primary analysis. Moreover, the relative order of mind–body and multimodal exercise changed after trials using regular physical-education controls were excluded (Supplementary Figure S4), indicating that lower- and middle-ranked comparisons were sensitive to the choice of control condition. Seventh, the P-score rankings should be interpreted with caution. The confidence intervals of the leading interventions overlapped substantially; therefore, the rankings reflect a relative ordering rather than definitive differences in effectiveness. Finally, the available data were insufficient to conduct subgroup analyses or meta-regressions examining the moderating effects of exercise dose parameters, such as frequency, intensity, session length, and total intervention duration.

Despite these limitations, the findings suggest that several exercise modalities may be useful for improving body image in women. Aerobic exercise and dance showed significant benefits relative to usual care, while multimodal exercise also produced a significant effect. Given the uncertainty in comparative rankings, intervention choice should be guided by feasibility, accessibility, participant preference, and the implementation setting rather than by P-score ranking alone. For individuals seeking varied experiences or pursuing multiple health-related goals, multimodal exercise offers a flexible and practical alternative. For those with high body dissatisfaction or prior negative exercise experiences, including a history of trauma or eating disorders, a trauma-informed approach emphasizing safety, autonomy, and functional goals may be appropriate. Appearance-focused exercise motives may be associated with less favorable body image outcomes, whereas more intrinsic or health-oriented motives may be associated with more adaptive responses (Panão and Carraça, 2020). Future research should prioritize adequately powered randomized controlled trials of under-represented modalities, standardized reporting of exercise dose parameters, the development of core outcome sets for body image assessment, and longer-term follow-up to evaluate the persistence of intervention effects beyond the active treatment period.

## 6 Conclusion

This systematic review and network meta-analysis provides comparative evidence to inform the selection and design of exercise interventions for improving body image in women. Among the evaluated modalities, aerobic exercise and dance showed the most consistent evidence of benefit across the primary and sensitivity analyses, while multimodal exercise also showed beneficial effects and resistance training showed a favorable but less precise effect. These findings support consideration of aerobic exercise and dance when designing exercise programs aimed at improving body image, while modality selection should also consider individual preferences, physical capacity, and implementation settings. However, the available evidence does not establish clear superiority of any single modality, and treatment rankings should therefore not be interpreted as a definitive hierarchy of effectiveness. Future research should prioritize adequately powered head-to-head randomized trials, more consistent assessment of body image, standardized reporting of exercise dose, and longer-term follow-up to refine exercise recommendations and determine the sustainability of intervention effects.

## Data Availability

The original contributions presented in the study are included in the article/Supplementary material, further inquiries can be directed to the corresponding authors.
